# Non-strabismic Binocular Vision Dysfunction among the Medical Students of a Teaching Hospital: A Descriptive Cross-sectional Study

**DOI:** 10.31729/jnma.7615

**Published:** 2022-08-31

**Authors:** Pooja Shrestha, Raju Kaiti

**Affiliations:** 1 Department of Ophthalmology, Kathmandu University School of Medical Sciences, Dhulikhel, Kavre, Nepal; 2 Department of Optometry, Nepal Eye Hospital, Tripureshwor, Kathmandu, Nepal

**Keywords:** *binocular vision*, *convergence excess*, *convergence insufficiency*, *prevalence*

## Abstract

**Introduction:**

Non-strabismic binocular vision dysfunctions are visual disorders that affect the person's binocular vision and visual outcome while performing near tasks and are very common among medical students. This study aimed to find out the prevalence of non-strabismic binocular vision dysfunction among the medical students of a teaching hospital.

**Methods:**

A descriptive cross-sectional study was conducted among medical students of a teaching hospital from 25 April 2022 to 25 May 2022. Ethical approval was obtained from the Institutional Review Committee of the same institute (Reference number: 139/17). A detailed ocular evaluation including history, visual acuity, refraction, and detailed orthoptic evaluation was done. Convenience sampling was done. Point estimate and 95% Confidence Interval were calculated.

**Results:**

Out of 284 students, 79 (27.81%) (22.60-33.02, 95% Confidence Interval) had non-strabismic binocular vision dysfunctions. Convergence insufficiency was the commonest one seen in 38 (48.10%), followed by divergence excess seen in 8 (10.12%) and convergence excess seen in 8 (10.12%) students.

**Conclusions:**

The prevalence of non-strabismic binocular vision dysfunction among medical students was lower than in other studies conducted in similar settings.

## INTRODUCTION

Non-strabismic binocular vision dysfunction (NSBVD) are visual disorders that affect the person's binocular vision and visual outcome when performing near tasks causing eye strain, headache, and blurred vision. If left undiagnosed or untreated, this defect can increase or turn into strabismic dysfunction.^1,2^ It can be classified as accommodative and vergence anomalies.^3^ These anomalies of binocular vision have been reported to be highly prevalent among school-going children and in adults too.^1,4^

Medical science is one of the highest levels of education requiring intensive near work. With the increasing prevalence of binocular vision anomalies and increasing demand for near work especially among medical students, timely diagnosis and intervention ensure good visual quality.^5^ But no studies have been conducted so far in Nepal assessing the binocular vision status among the medical students.

This study aimed to find out the prevalence of non-strabismic binocular vision dysfunction among the medical students of a teaching hospital.

## METHODS

A descriptive cross-sectional study was conducted among the medical students of Kathmandu University School of Medical Sciences, Dhulikhel, Kathmandu, Nepal from 25 April 2022 to 25 May 2022. Ethical approval was obtained from the Institutional Review Committee (Reference number: 139/17). Students who gave informed written consent were included in the study. Students with amblyopia, history of squint surgery, and use of any systemic or any ophthalmic drugs affecting binocular vision and accommodation were excluded from the study. Students from other allied medical fields like dental, nursing, and physiotherapy were also excluded. A convenience sampling method was used. The sample size was calculated using the following formula:


$$\begin{array}{ll} \text{n} & ={\text{Z}}^{2}\times \frac{\text{p}\times \text{q}}{{\text{e}}^{\text{2}}}\\ & =1.96^{2}\times \frac{0.315\times 0.685}{0.06^{2}}\\ & =231 \end{array}$$

Where,

n= minimum required sample size
Z= 1.96 at 95% Confidence Interval (CI)
p= prevalence of non-strabismic binocular vision dysfunction, 31.5%^4^
q= 1-p
e= margin of error, 6%

The minimum required sample size was 231. A 10% non-response rate was added and a sample size of 256 was obtained. However, a sample size of 284 students was taken. A detailed history was taken regarding the visual symptoms, and visual activities followed by a far and near visual acuity (VA) assessment. For far vision standard Snellen's chart at 6 meters and for near VA with reduced Snellen's chart at 50 cm were used. Both objective and subjective refraction was performed for all the students followed by the Worth-4-dot test, cover/uncover test at a distance and near, prism bar cover test, calculation of accommodative-convergence over accommodation (AC/A) ratio, and ocular motility test. Then, the binocular vision and accommodative assessments were done which includes measurement of near point of convergence (NPC) [with royal air force (RAF) rule], near the point of accommodation (NPA) (with RAF rule), phorias at distance and near, fusional vergence amplitudes using prism bar, monocular and binocular accommodative facility [with ±2.00 diopter sphere (DS) flipper]. AC/A ratio was calculated by the gradient method. The criteria for diagnosis of non-strabismic binocular vision anomalies were adopted from Scheiman and Wick.^6^

Data was collected by a qualified optometrist and supervised by the principal investigator ophthalmologist to ensure the quality of data and reduce bias.

Data were entered and analysed using Microsoft Excel 2011 and the IBM SPSS Statistics 11.5 respectively. Point estimate and 95% CI were calculated.

## RESULTS

Out of 284 students, 79 (27.81%) (22.60-33.02, 95% CI) students presented with non-strabismic binocular vision dysfunctions. Out of 79 students with NSBVD, 67 (84.81%) had vergence dysfunctions and 12 (15.18%) had accommodative dysfunctions (Table 1).

**Table 1:** Different types of binocular single vision anomalies (n= 79).

Binocular single vision anomalies	n (%)
Accommodative dysfunction	
Accommodation insufficiency	5 (6.32)
Ill sustained accommodation	2 (2.53)
Accommodation infacility	2 (2.53)
Accommodation excess	1 (1.26)
Pseudo-convergence insufficiency	2 (2.53)
Total	12 (15.81)
**Vergence dysfunction**	
Convergence insufficiency	38 (48.10)
Convergence excess	8 (10.21)
Divergence insufficiency	3 (3.79)
Divergence excess	18 (22.78)
Total	67 (84.81)

The students had a mean age of 22.98±1.80 years, with male predominance (1.82:1). A total of 51 (64.55%) students were males whereas, 28 (35.44%) were females. The mean Body Mass Index of the students was 23.00±15.48 with a mean height of 165.67±6.43 cm and a mean weight of 60.30±10.78 kg. The mean NPC was 8.00±2.35 and the mean near point of accommodation (NPA) was 9.89±1.67. The mean AC/A ratio was 6.22±0.27 (Table 2).

**Table 2:** Binocular vision parameters (n= 79).

Parameters	Mean±SD
AC/A	6.22±0.27
NPC (cm)	8.00±2.35
NPA (cm)	9.89±1.67
Accommodative facility cycle/min monocular	9.54±2.56
Accommodative facility cycle/min binocular	9.43±2.26

The mean hours of time spent by the students on study, computer/television and mobile phones were 3.90±1.96, 2.35±1.50 and 2.55±1.47 respectively.

## DISCUSSION

Non-strabismic accommodative and vergence dysfunctions are common vision anomalies encountered frequently and are usually associated with extensive near work. In this study, the prevalence of NSBVD was 27.81%. This finding is consistent with the study done in India, South Korea, Spain, and other countries where the prevalence was 29.60%, 28.52%, 22.26%, and 32.30% respectively.^4,7-10^ In contrast, other studies have reported an even higher prevalence of 54.66% to 73.69% of accommodative and vergence dysfunctions.^11,12^ This huge difference may be due to different population samples and different classification systems adopted.

In this study, vergence dysfunctions (84.81%) were higher compared to accommodative dysfunctions (15.81%). Likewise, a higher prevalence of vergence dysfunctions was detected than accommodative dysfunctions among the engineering students in Nepal and Optometry students in India which supports the findings of our study.^1,13,14^ Similarly, studies conducted in South Korea and Nigeria also revealed a higher prevalence of vergence dysfunctions than accommodative dysfunctions.^8,15^ However, in contrast, numerous studies in the past have concluded that accommodative dysfunctions are more prevalent than vergence dysfunctions.^1,10,16-19^ In this particular study, the sample population were medical students who are almost always exposed to near work which can play a role in the increased prevalence of vergence or binocular dysfunctions.

This wide range of prevalence of accommodation and vergence dysfunctions reported in the literature may be attributed to different criteria used for the diagnosis of various dysfunctions in different studies, type of population sample, and differences in sample size and also age group of the population.

Convergence insufficiency is the decreased ability to converge the eyes and maintain binocular vision while focusing on a near target and is caused by an imbalance of the vergence eye movements that are either inborn or acquired.^20^ In this study, the prevalence of vergence anomalies namely, convergence insufficiency has been predominantly reported similar to the studies conducted in several other countries.^4,8,14,21-24^ Similarly, a wide range of prevalence of covergence insufficiency between 2.25 to 33% has been reported in other studies which may be due to different diagnostic criteria used.^6,8,9,21-25^ Prevalence of remaining subtypes was reported less in this study and also there is a scarcity of literature regarding the other subtypes of anomalies. Furthermore, a significant association between study hours and non-strabismic anomalies of binocular vision has been reported in the literature.^26^ But this association could not be explored in this study due to the small sample size and design of the study.

This study has a few limitations. The sample size was small so it may not have covered all the anomalies and it is difficult to conclude that this sample may be representative of the whole university population and all the other study faculties. It demands extensive studies in the future comprising of a large population so that the results obtained can be implemented in preventive measures, awareness, or screening programs to detect these binocular vision anomalies early and interventions can be applied.

## CONCLUSIONS

The prevalence of NSBVD among medical students was lower than in previous studies done in similar settings. However, NSBVD could affect the academic performance of medical students hence a comprehensive ocular and optometric examination should be recommended in order to detect and treat binocular vision anomalies timely.
